# Efficient Generative-Adversarial U-Net for Multi-Organ Medical Image Segmentation

**DOI:** 10.3390/jimaging11010019

**Published:** 2025-01-12

**Authors:** Haoran Wang, Gengshen Wu, Yi Liu

**Affiliations:** 1 Faculty of Data Science, City University of Macau, Avenida Padre Tomás Pereira Taipa, Macao 999078, China; d24092100111@cityu.edu.mo; 2 School of Computer Science and Artificial Intelligence, Changzhou University, Changzhou 213000, China; liuyi0089@cczu.edu.cn

**Keywords:** image segmentation, medical image analysis, deep learning, attention mechanism

## Abstract

Manual labeling of lesions in medical image analysis presents a significant challenge due to its labor-intensive and inefficient nature, which ultimately strains essential medical resources and impedes the advancement of computer-aided diagnosis. This paper introduces a novel medical image-segmentation framework named Efficient Generative-Adversarial U-Net (EGAUNet), designed to facilitate rapid and accurate multi-organ labeling. To enhance the model’s capability to comprehend spatial information, we propose the Global Spatial-Channel Attention Mechanism (GSCA). This mechanism enables the model to concentrate more effectively on regions of interest. Additionally, we have integrated Efficient Mapping Convolutional Blocks (EMCB) into the feature-learning process, allowing for the extraction of multi-scale spatial information and the adjustment of feature map channels through optimized weight values. Moreover, the proposed framework progressively enhances its performance by utilizing a generative-adversarial learning strategy, which contributes to improvements in segmentation accuracy. Consequently, EGAUNet demonstrates exemplary segmentation performance on public multi-organ datasets while maintaining high efficiency. For instance, in evaluations on the CHAOS T2SPIR dataset, EGAUNet achieves approximately 2% higher performance on the Jaccard metric, 1% higher on the Dice metric, and nearly 3% higher on the precision metric in comparison to advanced networks such as Swin-Unet and TransUnet.

## 1. Introduction

Currently, the segmentation and annotation of organs in medical images are primarily performed manually [1,2]. Labeling lesions is a labor-intensive task, and the repetitive nature of this work, combined with low labeling efficiency, drains valuable medical resources. Therefore, there is a pressing need to design efficient algorithms capable of performing automatic segmentation. These algorithms can assist doctors in labeling and diagnosing lesions, enhance their work efficiency, and ultimately increase patient cure rates [3,4,5,6].

**Figure 1:**
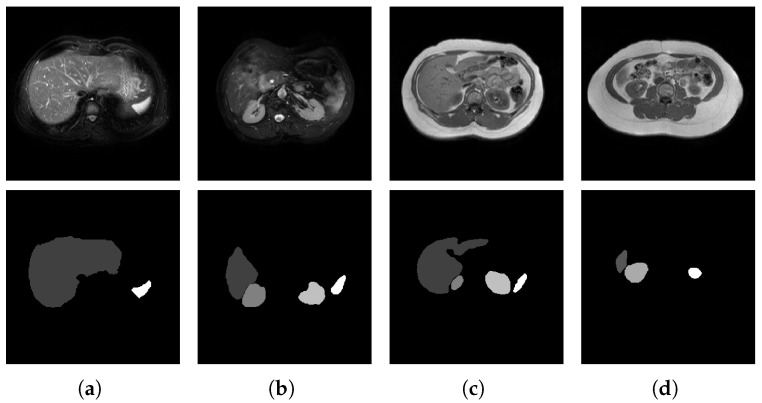
Segmentation results of different methods on CHAOS MR T2SPIR and T1DUAL datasets [20]. The first two columns for T2 SPIR and the others for T1DUAL. (**a**) T2SPIR image that includes the liver and spleen; (**b**) T2SPIR image includes the liver, right kidney, left kidney, and spleen; (**c**) T1DUAL image that includes the liver, right kidney, left kidney, spleen; (**d**) T1DUAL image that includes the liver, right kidney, and spleen.

The challenges in medical image segmentation include the limited availability of data samples, subtle key information, high similarity among medical images, and the multimodal nature of medical imaging [7,8]. Unlike natural images, where data collection and annotation are more straightforward, acquiring and annotating medical images requires the involvement of medical practitioners [6,9]. This manual annotation process is time-consuming and inefficient, resulting in a scarcity of high-quality annotated datasets [10,11]. In multi-organ segmentation tasks, dividing a medical image into regions that share similar properties (e.g., color, texture, brightness, or contrast) to accurately identify multiple organs or abnormal areas is challenging [12,13]. The annotated organs often occupy a small portion of the overall image, leading to a low signal-to-noise ratio. This emphasizes the need for algorithms that are sensitive to small targets. Additionally, human tissues exhibit minimal variation, causing significant similarities in medical images taken from the same anatomical location [14]. However, even minor changes can indicate the presence of lesions, necessitating that segmentation algorithms achieve high accuracy and effectively capture all relevant details in the image [7]. It is also important to note that medical images are captured using various imaging methods, including radiological imaging, functional imaging, magnetic resonance imaging (MRI), and ultrasound [15]. Each modality provides distinct information, and varying imaging parameters within the same modality can result in significantly different images [16]. For instance, Figure 1 displays abdominal MRI scans and labels acquired through different scanning methods. T1-weighted imaging (T1WI) highlights differences in the longitudinal relaxation of tissue T1 [17,18], making it useful for anatomical observation. Conversely, T2-weighted imaging (T2WI) focuses on differences in T2 transverse relaxation in tissues [19] and is particularly sensitive to bleeding, making it effective for detecting lesions. Consequently, designing a universal segmentation model that can accurately process diverse image types remains a significant challenge.

To summarize, problems such as blurred segmentation targets, an unpredictable number of segmentation targets, and low image contrast are common in medical image datasets [2,4,21]. Different scanning methods also result in varied image styles. A single patient can produce numerous images from MRI scans, necessitating efficient segmentation of multiple images to minimize the time needed for medical consultations. Thus, there is a critical need to develop neural networks that can effectively and accurately segment organ images from distinct scanning methods while addressing these challenges. In this work, we propose a novel deep framework termed Efficient Generative-Adversarial U-Net (EGAUNet) to perform automatic refined segmentation of medical images. By incorporating novel techniques like Global Spatial-Channel Attention (GSCA), Efficient Mapping Convolutional Block (EMCB), and a generative-adversarial learning strategy, it enables better segmentation results by engaging different information from conducting a large number of experiments on multi-organ, brain, and chest segmentation datasets. They show that the proposed method has excellent segmentation accuracy and robust generalization ability. The contributions of this article are as follows:

- Efficient Generative-Adversarial U-Net: We introduce a new medical image-segmentation framework designed for fast and accurate multi-organ segmentation. This model incorporates advanced feature processing modules that enhance the extraction of multi-scale spatial information and improve the model’s comprehension of this information. Additionally, the framework progressively refines these features using a generative-adversarial learning strategy, resulting in increased segmentation accuracy.
- Global Spatial-Channel Attention Mechanism: This mechanism enhances the model’s ability to perceive spatial information, enabling it to concentrate more effectively on specific areas of interest.
- Efficient Mapping Convolutional Block: This advanced block improves the network’s capability to gather multi-scale spatial information and uses a residual method to address issues associated with gradient descent and information loss.
- Training Strategy in Generative-Adversarial style: This proposed training approach significantly improves the prediction accuracy of the model generated using this technique. As a result, EGAUNet demonstrates superior segmentation performance compared to leading deep learning methods on publicly available multi-organ datasets, all while maintaining high efficiency.

In Section 2, some related works are briefly discussed. Then, we detail the proposed framework in Section 3 and present the experimental results in Section 4. Finally, this work is concluded in Section 5.

## 2. Related Works

In this section, we briefly discuss the applications of deep networks in multi-organ segmentation tasks and some generative-adversarial learning methods.

### 2.1. Classical Segmentation Networks in Medical Image Analysis

The distribution of segmentation targets in the human body is very regular, the semantics are relatively simple, and the low-resolution information can be used to identify the target object. Medical images have problems such as blurred boundaries and complex gradients, and high-resolution information can help models segment them more accurately. In 2015, Ronneberger et al. proposed the U-Net [22] network model, which is based on the encoder-decoder structure and applies long-distance hopping connections combined with the details from the bottom layer, which effectively makes up for the lack of spatial information in the process of downsampling operation and helps the network recover more accurate positioning. U-Net combines low-resolution and high-resolution information, making it a good fit for medical image segmentation. In 2018, Xiao et al. proposed Res-Unet [23] by replacing each submodule of U-Net with a form of residual connection and introducing an attention mechanism. In 2019, Zhou et al. proposed U-Net++ [24], which has the advantage of capturing features at different levels, integrating them through feature superposition, and adding a shallower U-Net structure so that the difference in the scale of the feature map during fusion is smaller. U-Net++ also introduces a lot of parameters and takes up more memory. A year later, Huang et al. proposed U-Net3+ [25], a full-scale hop connection that transforms the interconnection between encoders and decoders and the inline between decoder subnetworks. In 2019, Jha et al. proposed ResUNet++ [26] is based on ResUNet and continues to introduce extrusion and incentive mechanisms, ASPP and self-attention modules [27]. The following year, Double U-Nets [28] were proposed, and the network had two U-Nets. The encoding layer of the first U-Net and the decoding layer of the second U-Net are connected by hopping, and there is a multi-head transition operation, and the final output of the network is spliced by the production of the two U-Nets. In 2019, Ibtehaz et al. proposed MultiResUnet [29], which replaces the traditional convolution module in U-Net with the MutiRes module. Replace the simple hop connections in traditional U-Net with ResPath. In 2020, Lou et al. proposed DC-U-Net [30], which replaces the MultiRes module in MultiResUnet with a DC block. The authors believe that multi-scale information is conducive to the improvement of segmentation accuracy, and the information provided by a MultiRes block is not rich enough. In 2021, Chen et al. combined Transform [31] and U-Net to propose TransUnet [32]. Cao et al. replaced the convolutional block with a Win convolution block and proposed Swin-Unet [33].

Many scholars have proposed segmentation networks with various structures. Zhao et al. introduced the Pyramid Pooling Network (PSPNet) [34], which integrates global context information. Lin et al. developed the Feature Pyramid Network (FPN) [35], addressing issues of information loss and resolution mismatch during segmentation at different scales. Chen et al. presented DeepLabV3 [36], which enhances Atrous Spatial Pyramid Pooling by employing large samples of dilated convolutions. Subsequently, they introduced a new encoder-decoder structure, DeepLabV3+ [37], that uses DeepLabV3 as the encoder module. Additionally, Chaurasia et al. proposed LinkNet [38], which improves speed while maintaining accuracy. Lastly, Li et al. introduced PAN [39], which expands upon the FPN concept and enhances feature fusion methods.

In terms of organ segmentation, Roth et al. used a 3D fully convolutional network to segment multi-organ images [40]. Gibson et al. proposed DenseVNet [41] to achieve high-resolution segmentation through efficient memory loss and feature reuse and segmented 8 organoids in the abdominal dataset to demonstrate the effectiveness of the model. Wang et al. proposed a network for multi-organ segmentation to improve segmentation accuracy by adding back-connected organ attention [42]. Fan et al. proposed MA-Net [43]. A self-attention mechanism is introduced to adaptively integrate local features with their global dependencies. Lei et al. proposed SGU-Net [44], designed an ultra-light convolution that can realize double separable convolutions at the same time, and used an additional adversarial shape constraint to let the network learn the shape representation of the target, which significantly improved the segmentation accuracy of abdominal medical images.

### 2.2. Generative-Adversarial Learning Methods

In 2014, Goodfellow et al. proposed a Generative-Adversarial Network (GAN) based on probability and statistical theory through the perspective of game theory [45]. Luc et al. applied GAN to the field of image segmentation for the first time [46]. Yu et al. proposed SeqGAN [47] to process discrete sequence data using a generative-adversarial network. Mirza et al. proposed a CGAN conditional generative-adversarial network [48], which uses real labels as auxiliary information and adversarial networks that extend valid information to arbitrary available information. Isola et al. proposed that Pix2PixGAN [49] can learn the mapping from the input image to the output image using the input image as a condition. Odena et al. proposed a generative-adversarial network model based on semi-supervised learning, SGAN [50], in which the real data are labeled, and the data generated by the generator are unlabeled. Zhu et al. proposed CycleGAN [51], which transforms images from one domain to another by training two pairs of generator and discriminator models. The super-resolution generative-adversarial network SRGAN [52] proposed by Ledig et al. realizes the generation of low-resolution images into high-resolution images by generating adversarial networks without distortion. The ESRGAN [53] proposed by Wang et al. removes all the batch normalization layers of the generator and uses the relative discriminator as the discriminator, allowing the discriminator to estimate the probability that the real image is more realistic than the generated image.

## 3. Proposed Method

### 3.1. Overall Framework of EGAUNet

**Figure 2:**
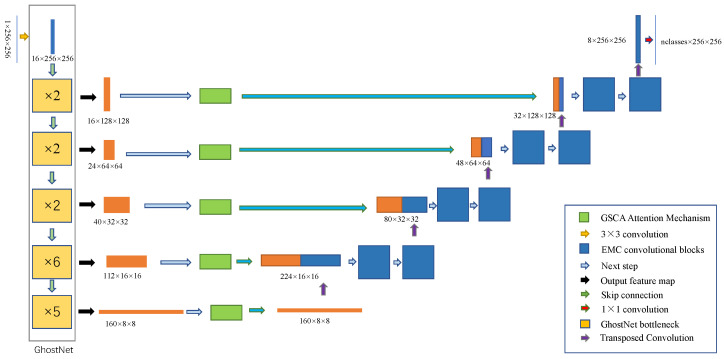
The encoder-decoder structure of the proposed EGAUNet. The encoder section on the left is based on GhostNet’s bottleneck architecture. In the center, there is a skip connection where the Global Spatial-Channel Attention is implemented. On the right, the decoder module is composed of Efficient Mapping Convolutional Blocks.

This section provides a detailed overview of the proposed EGAUNet by thoroughly analyzing the various submodules and learning strategies. Key components include the Efficient Mapping Convolutional Block, the Global Spatial-Channel Attention mechanism, and Generative-Adversarial Training. Figure 2 illustrates the structural diagram of the framework. The entire system is divided into three main parts: the feature extraction module (i.e., Section 3.2), the decoder module (i.e., Section 3.3), and the generative-adversarial learning strategy (i.e., Section 3.4).

### 3.2. Feature Extraction Module

The feature extraction module, also known as the encoder, analyzes the input image to obtain deep information. It reduces the resolution of the feature map through a series of convolution and pooling operations, allowing it to capture essential details. This module is based on GhostNet [54] and incorporates a proposed Global Spatial-Channel Attention Mechanism.

#### 3.2.1. GhostNet Bottleneck Layer

**Figure 3:**
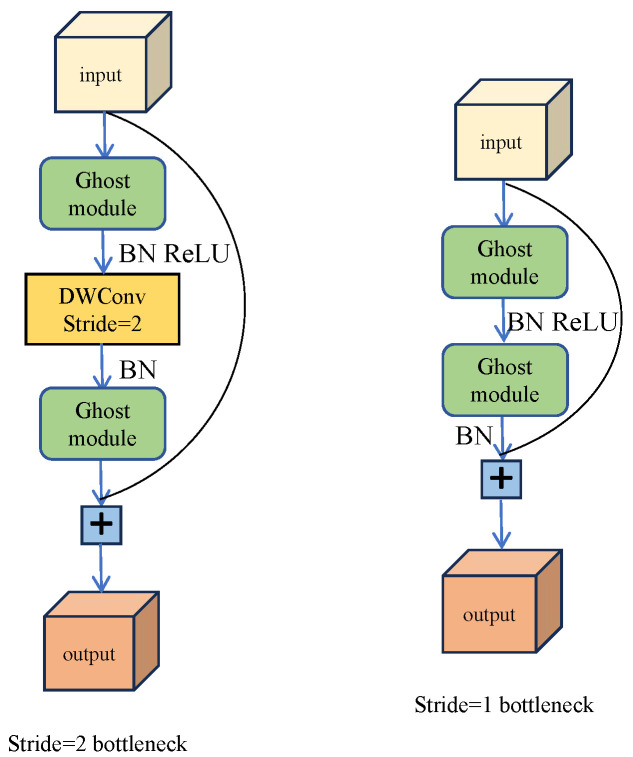
The flow diagram of GhostNet bottleneck layer. When the stride is 1, it is connected by two ghost modules and residuals. When the stride is 2, one more Depthwise-Separable Convolution is performed.

In this module, GhostNet [54] is chosen as the backbone model. It leverages transfer learning by utilizing GhostNet’s pre-trained weights on the ImageNet dataset. Compared to random initialization weights, using pre-trained weights allows the model to converge faster and achieve optimal solutions more efficiently. Experimental results show that GhostNet can provide the same or higher accuracy as other lightweight models such as MobileNet [55] and ShuffleNet [56] while maintaining computational efficiency. GhostNet has 50% fewer parameters and computations than MobileNetV3 [57], but the performance is comparable. The design incorporates concepts from MobileNetV2 [58], notably omitting the ReLU activation function in the last two layers of batch normalization, which reduces computational effort. A key component of the GhostNet bottleneck layer is the ghost convolution blocks. Ghost convolution is based on the principles of convolution techniques found in models like MobileNet [55] and ShuffleNet [56]. Figure 3 illustrates the flow diagram of the bottleneck layer in GhostNet. When a feature map is input into GhostNet, a 1×1 convolution is performed to make the number of channels of the feature map 16. After that, the feature map is input into the stacked bottlenecks. The number of ghost bottlenecks stacked is 2, 2, 2, 6, and 5 in order. The number of channels of the obtained feature map is 16, 24, 40, 112, and 160, respectively. The size of the feature map is changed from the bottleneck of Stride=2 in Figure 3 to 1/2 of the previous layer.

#### 3.2.2. Global Spatial-Channel Attention

In the field of computer vision, most neural network architectures rely on local feature extraction methods. These methods focus solely on the correlations within a small section of the image contained in the receptive field, often neglecting the extraction of global features. Conventional convolutional neural networks are limited by their local perception. To capture long-range information, these networks typically require stacking multiple convolutional layers, which can lead to low training efficiency, challenging information transmission, and difficulties in optimizing the network.

**Figure 4:**
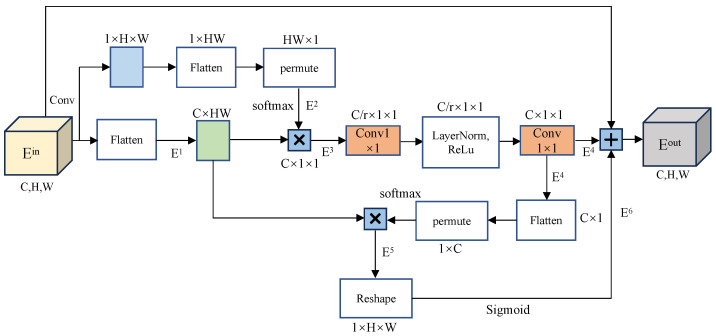
The structure of the proposed GSCA. The dimensional transformation of the feature map, matrix multiplication and other operations improve the perception ability of the Global Spatial-Channel Attention mechanism for spatial information.

To address this issue, this paper proposes a Global Spatial-Channel Attention (GSCA) mechanism and applies it to the extracted features from these GhostNet bottleneck layers, as shown in Figure 2. The local channel attention mechanism serves primarily as a context modeling module that aggregates features from all locations to create a global context feature. Additionally, it includes a feature transformation module designed to capture interdependencies between channels. Furthermore, there is a fusion module that integrates the global context into every location in the image. Through context modeling, a global information relationship vector is obtained. Subsequently, a two-layer 1×1 convolution is applied to reduce the number of parameters and further extract relevant information. Finally, the spatial feature extraction component enables the model to focus more on regions of interest by assigning weights to each pixel. Figure 4 illustrates the structure of the proposed GSCA, and the specific calculations of this module are illustrated below.

First, the input feature map $E^{in}$ is flattened to obtain the feature map $E^{1}=Flatten\left(E^{in}\right)$. The dimension of $E^{1}$ is C×HW. At the same time, the input feature map $E^{in}$ is input into the 1×1 convolution, and the feature map $E^{2}$ is obtained by channel transformation and other operations and input into SoftMax for normalization, namely $E^{2}=\sigma \left(Permute\left(Flatten\left(Conv\left(E^{in}\right)\right),{dim}_{1}\right)\right)$, where Permute represents the channel transformation and $dim_{1}\in \left[0,2,1\right]$ represents the order of the new dimension. The dimension of $E^{2}$ is HW×1. Then $E^{1}$ and $E^{2}$ are multiplied by matrices, and the feature map was reshaped into a new shape, namely $E^{3}=view\left(E^{1}\times E^{2}\right)$, where view represents shape remodeling and the dimension of $E^{3}$ is C×1×1.

The feature map $E^{3}$ is further processed by an 1×1 convolution and layer normalization (i.e., $Conv_{1\times 1}$ and LN as $E^{4}=Conv_{1\times 1}\left(LN\left(Conv_{1\times 1}\left(E^{3}\right)\right)\right)$, which yields $E^{4}$ with the size of C×1×1. After passing the Flatten and Permute operations of feature map $E^{4}$, the shape of the feature map is changed to 1×C, and then input into SoftMax (i.e., σ(·)) to assign a weight value to each channel. The input feature map is flattened to change the shape from C×H×W to C×HW, and $dim_{2}\in \left[1,0\right]$ represents the order of the new dimension. In addition, then the two are multiplied by the matrix to obtain a 1×HW feature map $E^{5}=\sigma \left(Reshape\left(Flatten\left(E^{4}\right),{dim}_{2}\right)\right)\times Flatten\left(E^{in}\right)$.

The shape size of the a-matrix is 1×C, and the size of the c-matrix is 1×HW obtained by multiplying the a-matrix with the weight of each channel and the b-matrix with the shape size C×HW. The c matrix is transformed to obtain 1×H×W, as shown in the following matrix:

(1) $$\left(\begin{array}{c} \begin{array}{cccc} a_{1} & a_{2} & \ldots & a_{C} \end{array} \end{array}\right)\left(\begin{array}{c} \begin{array}{cccc} b_{111} & b_{112} & \ldots & b_{1HW}\\ b_{211} & b_{212} & \ldots & b_{2HW}\\ \vdots & \vdots & \ddots & \vdots \\ b_{C11} & b_{C12} & \ldots & b_{CHW} \end{array} \end{array}\right)=\left(\begin{array}{c} \begin{array}{cccc} c_{11} & c_{12} & \ldots & c_{HW} \end{array} \end{array}\right),$$

The *c* matrix is transformed to obtain 1×H×W, as shown in the following matrix:

(2) $$\left(\begin{array}{cccc} c_{11} & c_{12} & \ldots & c_{1W}\\ c_{21} & c_{22} & \ldots & c_{2W}\\ \vdots & \vdots & \ddots & \vdots \\ c_{H1} & c_{H2} & \ldots & c_{HW} \end{array}\right).$$

The multiplication operation assigns different values to each pixel. $E^{5}$ is followed by reshape operation and SoftMax operation to obtain $E^{6}=\sigma \left(Reshape\left(E^{5},\left(d_{1},d_{2},d_{3}\right)\right)\right)$, where *d* represents the dimension in which the feature map would be reshaped. σ represents that the feature map is input into the Softmax function. Then the feature map output after GSCA (i.e., $E^{out}$) can be obtained by summing $E^{in}$, $E^{4}$, and $E^{6}$ up as $E^{out}=E^{in}+E^{4}+E^{6}.$

By doing so, it enhances the model’s focus on the target region by increasing the weight of that area. Simultaneously, the weight of the background pixel positions is reduced, minimizing the interference of the background during model training. This weighting strategy optimizes the model’s resource allocation across different regions, allowing it to concentrate more on the features of the target area during training. Furthermore, by incorporating a spatial attention mechanism, the model can further enhance its attention on the target area. The combination of weight adjustment and the spatial attention mechanism significantly improves the neural network’s ability to extract features from the region of interest. Ultimately, this approach enhances the model’s segmentation accuracy in complex scenes, particularly when dealing with small targets, blurred boundaries, or low contrast between the target and the background.

### 3.3. Decoder Module

After the encoding process, the decoder module is launched to convert the low-resolution feature map back into a high-resolution feature map, and combine it with the coded feature map at the same scale.

#### Efficient Mapping Convolutional Block

**Figure 5:**
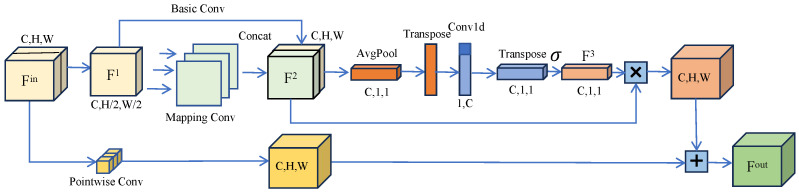
Efficient Mapping Convolutional Block. The features of the feature map are extracted through three parts: basic convolution, mapping convolution, and point-by-point convolution, and the weight value of each channel of the feature map is given through dimensional transformation.

This module introduces an Efficient Mapping Convolutional Block (EMCB), and its workflow is illustrated in Figure 5. Initially, the number of channels in the feature map is reduced by half using a basic convolution. This approach results in fewer feature maps than those found in other neural networks, therefore decreasing the model’s computational cost. Next, the output feature map is processed through channel-by-channel convolution for linear mapping, which generates a graph similar to the input feature map and increases the number of feature maps. Finally, the output feature map is concatenated with the input feature map along the channel dimension. This method of convolution has nearly half the number of parameters compared to standard convolution. The input feature map $F^{in}$ undergoes a convolution with a 3×3 filter (i.e., $Conv_{3\times 3}$), which results in a new feature map $F^{1}$ with half the number of channels, namely $F^{1}=Conv_{3\times 3}\left(F^{in}\right)$.

Next, after applying deep convolution, $F^{1}$ is concatenated with itself along the channel dimension to produce $F^{2}=Concat\left[F^{1},DConv\left(F^{1}\right)\right],$ where DConv denotes deep convolution. During the initial convolution, the number of channels in the feature map is halved, which can lead to the loss of important information that might exist in those discarded channels. Furthermore, the feature map generated after the deep convolution operation is based on a nonlinear transformation of the already reduced channels, making it impossible to recover the discarded, important features during the concatenation process. To address these issues, this work employs the concept of residuals to transform the dimensions of the original feature map using channel convolution. By incorporating attention weighting in the addition of feature maps, the method aims to retain the valuable information contained in the original feature map, namely $F^{3}=\sigma \left(UST\left(Conv1d\left(ST\left(Avgpool\left(F^{2}\right)\right),pos\right)\right),pos\right)$. Avgpool refers to global average pooling, while ST denotes operations such as squeeze and transpose. Additionally, UST indicates operations like unsqueeze and transpose. The term Pos represents changes in channel positioning, where pos∈[−1,−2] means that the last two dimensions of the feature map are exchanged. Initially, the feature map $F^{2}$ undergoes global average pooling, resulting in a feature map with dimensions of C×1×1. Through operations like squeeze and transpose, this feature map is transformed into a one-dimensional format of 1×C. Following this, a one-dimensional convolution (i.e., Conv1d) is applied to capture dependencies between channels. Afterward, the feature map is reshaped back to the size of C×1×1 through channel reordering and dimension enhancement. Using $k=\left\lceil \log_{2}\left(C\right)\right\rceil +1$, the kernel size *k* for the one-dimensional convolution is calculated, where *C* represents the number of input channels, and ⌈·⌉ indicates a rounding-up operation. Adding 1 after rounding ensures that the minimum value of *k* is 3, which helps maintain an effective interaction range between channels. The value of *k* is correlated with the number of channels *C* through a dynamic formulation, allowing for an adaptive range of channel interactions at different model scales.

Then, the feature maps $F^{2}$ and $F^{3}$ undergo a Sigmoid activation, and then they are multiplied to assign a weight value to each channel of the feature map. This process retains the integrity of the original channel features, helping the model better utilize the dependencies between channels and enhancing its feature representation capability. Additionally, the feature map is processed through convolution on a channel-by-channel basis, where the feature weights assigned are summed up, therefore preserving the information from the original feature map, namely $F^{out}=F^{2}\cdot F^{3}+PConv\left(F^{in}\right)$. Here, PConv refers to pointwise convolution. This approach enhances the important channels that the original convolution module might overlook, ultimately improving the feature extraction capability of the convolution block. $F^{out}$ represents the output feature map after EMCB.

### 3.4. Generative-Adversarial Learning Strategy

**Figure 6:**
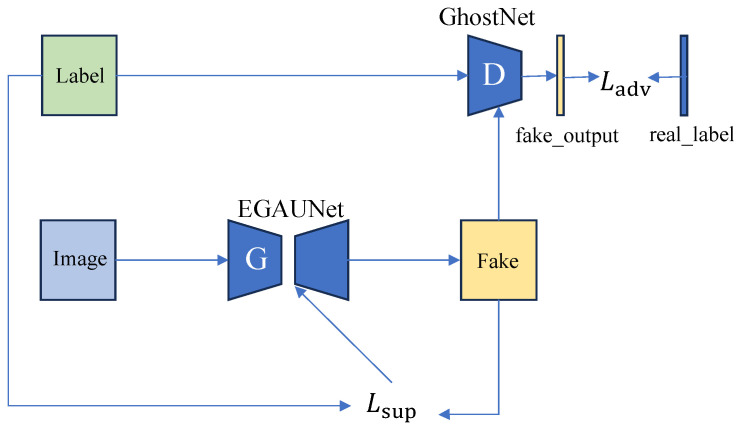
The generative-adversarial learning strategy utilizes GhostNet as the discriminator and the EGAUNet as the generator. The loss function consists of two components. The first component, denoted as $L_{sup}$, is the supervised term based on the Dice loss function. The second component, $L_{adv}$, serves as the adversarial term, which is used to adjust the loss for the discriminator.

Inspired by the concept behind Generative-Adversarial Networks (GAN) [45], we further implement a generative-adversarial learning strategy following the autoencoder in the proposed EGAUNet. This approach enhances the production of more refined segmentation results. In this framework, EGAUNet functions as the generator (i.e., *G*), while GhostNet serves as the discriminator (i.e., *D*). This combination improves the quality and detail of the segmentation outcomes through a seamless integration of U-Net and GAN architectures. By enabling the generator to create finer segmentation results, the discriminator can guide the generator toward producing more realistic outcomes. The adversarial interaction between the generator and discriminator fosters mutual improvement, ultimately leading to superior segmentation results. A detailed structural design is illustrated in Figure 6. In particular, the formula for GAN is illustrated as follows: $\begin{array}{c} \min_{G}\;\max_{D}\;V\left(D,G\right)=E_{x∼P_{data(x)}}\log D(x)+E_{z∼P_{Z(z)}}\left[\log\left(1-D(G(x))\right)\right]. \end{array}$

Let the value of D(x) approach 1 while simultaneously enhancing the discriminator’s ability to differentiate between real and fake data generated by the generator. Specifically, we aim to make D(G(x)) approaches 0. This leads us to maximize the function V(D,G). When updating the generator’s weights, we focus on training the generator while keeping the discriminator fixed. The goal is to make the generated images increasingly resemble real images, even as D(G(x)) approaches 1. By substituting into Equation, we minimize V(D,G), which can ultimately be simplified to a constant plus the Jensen-Shannon Divergence (JSD) after breaking down the formula. By optimizing $\min_{G}\;-2\log2+2JSD\left(P_{data}\left|\right|P_{G}\right),$ $P_{data}$ and $P_{G}$ will eventually be equal, the JSD value is the smallest, i.e., the generated sample is infinitely approximated with the real sample so that the distribution of the generated sample is similar to the distribution of the real sample.

### 3.5. Loss Function

This study focuses primarily on multi-organ segmentation, which is essentially a multi-class segmentation task. To effectively identify and accurately segment different organs, the overall loss function $L_{total}$ of the proposed framework comprises two components. The first component is the supervised term $L_{sup}$, which is based on the Dice loss function and calculated as below:

(3) $$L_{sup}=E_{x,y∼X_{L}}\left[CE\left(y,f_{\theta }\left(x\right)\right)\right],$$

where CE, *y*, f(·), and θ refer to the cross-entropy, ground truth, deep network and its parameters, respectively. The second component is the adversarial term $L_{adv}$, which adjusts the loss for the discriminator *D*.

(4) $$L_{adv}=-E_{x∼X}\left[\log\left(D\left(f_{\theta }\left(x\right)\right)\right)\right],$$

This term employs a binary cross-entropy loss function to penalize the discriminator for misclassifying real images and the segmentation maps produced by the generator. By considering the above equations, the overall loss function is presented:

(5) $$L_{total}=L_{sup}+\lambda L_{adv}.$$

where λ is the balancing parameter and λ=0.01 in the experiments.

## 4. Experiment and Discussion

In this section, extensive experiments are performed on public datasets to verify the performance of the proposed method and the results are implicitly discussed.

### 4.1. Datasets

The CHAOS (Combined CT-MR Healthy Abdominal Organ Segmentation) multi-organ segmentation dataset consists of two distinct parts: the CHAOS T2SPIR dataset and the CHAOS T1DUAL dataset [20]. The CHAOS T2SPIR dataset includes 503 training images and 120 test images. In contrast, the CHAOS T1DUAL dataset contains 530 training images and 117 test images. To assess the generalization of our model, we also selected two additional datasets. The first is the Brain MRI dataset, which includes brain MR images along with manual FLAIR abnormality segmentation masks [59,60]. These images were obtained from The Cancer Imaging Archive (TCIA) and correspond to 110 patients included in The Cancer Genome Atlas (TCGA). This dataset comprises 3000 training images and 929 test images. The second additional dataset is the Chest X-ray Masks and Labels dataset. It contains X-ray images along with their corresponding masks. This dataset includes 564 training images and 140 test images [61,62].

### 4.2. Implementation Details

This experiment is implemented using the PyTorch framework, PyCharm as the compilation platform, Intel (R) Xeon (R) Platinum 8255C CPU, RTX-2080Ti GPU with 11 GB of video memory, CUDA version 11.0, Linux operating system, and Python compilation language. The format of the CHAOS dataset is DICOM format, and we use PyDicom to read the dataset and change the original image to a size of 256×256. The pixel values in the label diagram are divided into five categories: 0, 1, 2, 3, and 4 according to the liver, right kidney, left kidney, and spleen. The specific settings for each layer of convolution blocks in EGAUNet are shown in Table 1. The Adam optimizer is used to update the network weights, the initial learning rate is set to 0.001, and the multi-step attenuation strategy (MultiStepLR) is adopted, the training rounds are set to 100, and the number of images input for each training session is set to 8. To compare with other networks to prove the effectiveness of this experiment, Accuracy, Jaccard coefficient, Recall, Dice, and Precision are selected as evaluation indicators. For the generative-adversarial training, we update the discriminator’s parameters once every 30 iterations and set the hyperparameter λ in the generator’s adversarial loss to 0.01.

### 4.3. Experimental Results on Diverse Datasets

Several mainstream segmentation networks such as U-Net [22], U-Net++ [24], DeepLabV3 [36], DeepLabV3+ [37], FPN [35], PAN [39], PSPNet [34], MA-Net [43], LinkNet [38], TransUnet [32], Swin-Unet [33], etc., are selected as baselines in the experiments.

#### 4.3.1. CHAOS T2SPIR Dataset

**Figure 7:**
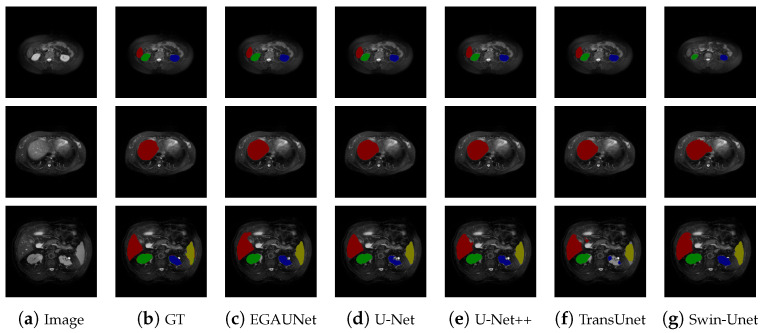
Segmentation results of different methods on the CHAOS T2SPIR dataset.

The results of various indicators from the experiment are shown in Table 2. Notably, EGAUNet outperforms other mainstream segmentation networks in terms of Accuracy, Jaccard index, Dice coefficient, and Recall, demonstrating its advantages. For example, compared to U-Net, the values achieved by EGAUNet are around 1.3%, 0.8%, and 2% higher on Jaccard, Dice, and Recall, respectively. It is also worth mentioning that EGAUNet remains a reasonable model size (e.g., 85% less than U-Net) when performing accurate segmentation, which implies its high efficiency. In the segmentation results of Figure 7, the red-highlighted organ represents the liver, the green organ is the right kidney, the blue organ is the left kidney, and the yellow organ is the spleen. In the first input multi-organ image, despite the small volume of each organ, EGAUNet trained with generative-adversarial methods aligns more closely with the label map than the other comparison networks when segmenting the liver. In the second multi-organ image, EGAUNet’s segmentation shape is more consistent with the label diagram. The third multi-organ image presents a more challenging segmentation scenario, featuring various organs of different shapes and sizes, along with some noise from the MRI data. Unlike other segmentation networks, EGAUNet achieves clean segmentation without multiple segmentations, misclassifications, or redundant pixels. This reflects the high predictive accuracy of the EGAUNet network. From the analysis and segmentation result graphs, it is evident that EGAUNet outperforms other networks in single-class and multi-class images, resulting in a lower probability of false predictions.

#### 4.3.2. CHAOS T1DUAL Dataset

**Figure 8:**
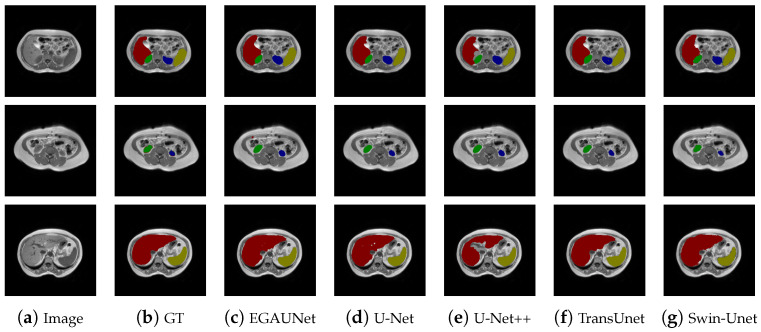
Segmentation results of different methods on the CHAOS T1DUAL dataset.

To verify the generalization of the EGAUNet network, which was trained using generative-adversarial training, we conducted tests on the CHAOS T1DUAL dataset. The T1DUAL and T2SPIR datasets utilize different weighted imaging techniques. The T2 signal is indicative of water content, highlighting many lesions and allowing for a clear identification of the location and size of the organs. In contrast, the T1 signal does not emphasize the organs as much, as it primarily focuses on the anatomical structure. We used 530 images from the T1DUAL dataset as the training set and 117 images as the test set, employing data augmentation during training. The results of the test data comparison are summarized in Table 3. From Table 3, it is evident that EGAUNet outperforms other mainstream comparison networks in terms of Accuracy, Dice coefficient, and Recall after undergoing generative-adversarial training. EGAUNet’s Jaccard index is slightly lower than that of DeepLabV3, particularly in the context of medical image segmentation experiencing significant class imbalances. In these situations, the Dice coefficient is generally considered more robust, while the Jaccard index is used as a supplementary comparison metric. However, in other datasets, EGAUNet’s Jaccard index surpasses that of DeepLabV3. Overall, these results indicate that EGAUNet performs better than DeepLabV3. Moreover, significant improvements in accuracy, Jaccard index, Dice coefficient, recall, and other metrics were observed when compared to the original U-Net network. Figure 8 presents a comparison of the segmentation results from various networks on the CHAOS T1DUAL dataset. As illustrated in the figure, EGAUNet achieves better segmentation results on the CHAOS T1DUAL dataset following generative-adversarial training.

#### 4.3.3. Brain MRI Dataset

**Figure 9:**
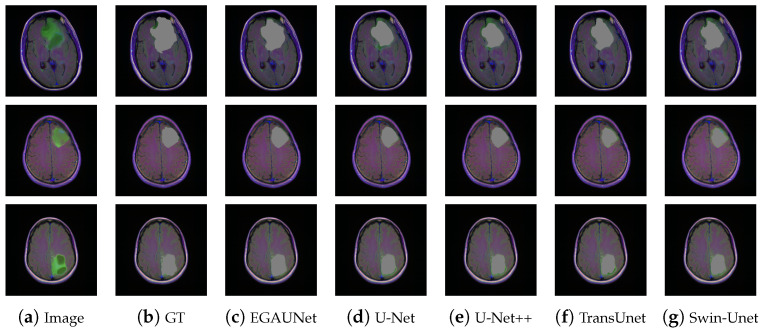
Segmentation results of different methods on the Brain MRI dataset.

In this work, we continued to perform experiments in Brain MRI segmentation. The experimental data are shown in Table 4. Table 4 shows the segmentation values of each model on the Brain MRI dataset. As can be seen from the table, EGAUNet outperforms the comparison network in all indicators. Among them, the Recall indicator is about 3% higher than that of U-Net. Figure 9 shows the segmentation results of each model on the dataset. It can be seen that EGAUNet can segment the lesion area more accurately than other networks in the segmentation results.

#### 4.3.4. Chest X-Ray Masks and Labels Dataset

**Figure 10:**
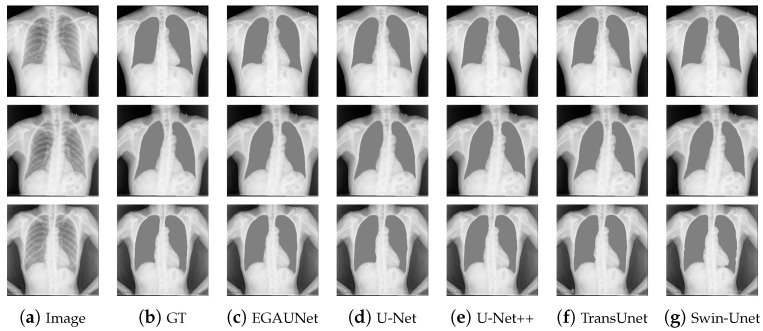
Segmentation results of different methods on the Chest X-ray Masks and Labels dataset.

Table 5 shows the segmentation values for each model on the Chest X-ray Masks and Labels dataset. As can be seen from the table, EGAUNet outperforms other comparison networks on the Jaccard and Dice indicators, which further validates the claimed contributions from the side. Compared to U-Net++, EGAUNet is 0.1% higher on Jaccard, 0.07% higher on Dice, and 0.3% higher on Recall. Figure 10 shows the segmentation results of each model on the dataset, and it can be seen that EGAUNet also shows good segmentation results.

### 4.4. Ablation Study

#### 4.4.1. Results from Different Attention Mechanisms

To investigate the effectiveness of the proposed GSCA, we conducted experiments by incorporating different attention mechanisms into the skip connection section. The experimental data are presented in Table 6. The abbreviations used are as follows: CBAM (Convolutional Block Attention Module) [63], GCNet (Global Context Network) [64], GAM (Global Attention Mechanism) [65], CCNet (Criss-Cross Network) [66], and SCSE (Concurrent Spatial and Channel Squeeze and Excitation) [67]. As shown in the table, the proposed GSCA outperforms the other attention mechanisms in terms of the Jaccard index, Dice coefficient, and accuracy. Although the recall indicator is slightly lower than that of CBAM, all those results admit the strong information extraction capability of GSCA.

#### 4.4.2. Results from Diverse Modules

**Figure 11:**
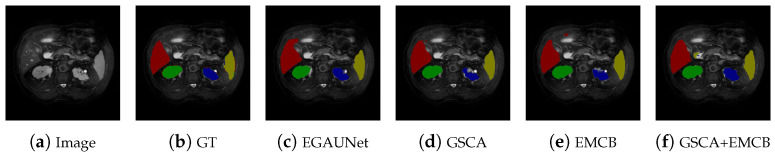
Segmentation results of different modules in the proposed framework. EGAUNet means a complete model with all modules added. GSCA indicates that only the Global Spatial-Channel Attention mechanism has been added. EMCB means that only the Efficient Mapping Convolutional Block has been added. GSCA+EMCB means that both the Spatial-Channel Attention mechanism and the Efficient Mapping Convolutional Block are added.

Table 7 presents the results of ablation experiments conducted on various advanced modules within the proposed framework, including GSCA and EMCB. Specifically, GAL refers to the Generative-Adversarial Learning strategy. The table shows that when GSCA and EMCB are incorporated individually, there is an improvement in all performance indicators. Furthermore, when both GSCA and EMCB are combined, the indicators show even greater enhancements. After the implementation of the Generative-Adversarial Learning training method, the model achieves its highest performance metrics. Notably, the Jaccard and Precision indicators have significantly improved compared to the initial network, and other metrics have also shown positive advancements. The modules developed and refined in this section effectively enhance the model’s performance. Figure 11 represents the segmentation comparison chart of each stage of the ablation experiment. As can be seen from the figure, the final model (i.e., (c)) performs better than simply adding a single module (i.e., (d), (e)) or two modules (i.e., (f)).

#### 4.4.3. Hyperparameter Analysis

In this section, we investigate the performance variations when selecting different λ from the loss function on the CHAOS T2SPIR dataset. The experimental results are shown in Table 8. As can be seen, the model results are higher than those of Accuracy, Jaccard, Dice, Recall, and Precision when λ=0.01, which is consistent with the optimal selection in the experiments.

#### 4.4.4. Effect of Residual Connections

To mitigate the potential loss of important information caused by halving the number of channels in the feature map during the initial convolution, we incorporate residual connections into the EMCB. We conducted ablation experiments to evaluate the impact of these residual connections, as illustrated in Table 9. Our comparison of EGAUNet’s performance with and without these residual connections demonstrates that EGAUNet with residual connections in the EMCB outperforms the version without them.

### 4.5. Computational Costs

In this section, we compare the computational costs of each model, focusing on Model Size, Giga Floating-point Operations Per Second (GFLOPs), and iterations per second (iter/s). The results are presented in Table 10. The model size and GFLOPs of EGAUNet are significantly lower than those of the other comparison networks. Additionally, EGAUNet performs competitively against PSPNet in terms of iterations per second, which implies its high efficiency.

### 4.6. Discussion

This section details a comprehensive series of experiments conducted to evaluate the performance of the proposed EGAUNet network across several datasets. The experimental results demonstrate that EGAUNet achieves commendable performance on all three datasets while also maintaining a model size that is notably smaller than that of comparable networks in the field. To gain deeper insights into the contributions of each module within the network, a set of ablation experiments was performed. These experiments highlighted that each module significantly enhances the overall accuracy of the model, reinforcing the effectiveness of the design choices made in the network’s architecture. Despite these positive outcomes, certain limitations of the model were identified. In particular, the segmentation accuracy for the CHAOS T1DUAL dataset was found to be lacking, suggesting a need for additional optimization and adjustments. Furthermore, there are opportunities for refining the model in terms of its size, computational demands, and operational speed, thus paving the way for future enhancements that could improve its efficiency in practical applications.

## 5. Conclusions

This paper introduces EGAUNet, an efficient and lightweight neural network specifically designed for medical image-segmentation tasks. In the feature processing stage, we have integrated innovative modules known as GSCA and EMCB. These modules substantially enhance the quality of representation for both encoded and decoded feature maps, leading to improved segmentation accuracy. Additionally, we employ a generative-adversarial learning framework following the feature-learning phase. This methodology facilitates continuous adversarial training between two interconnected networks, resulting in a significant enhancement of EGAUNet’s segmentation performance. When compared to various leading medical image-segmentation networks, EGAUNet demonstrates a noteworthy reduction in parameter count while also enhancing operational speed. Furthermore, the segmentation results generated by EGAUNet are comparable to, and in some instances, superior to, those obtained from existing models. The proposed segmentation network, EGAUNet, has the potential to effectively assist healthcare professionals in making informed clinical decisions, therefore greatly improving their operational efficiency. In our future research, we intend to further optimize segmentation performance by exploring techniques such as network pruning to eliminate unused or less critical parameters, as well as parameter binarization to reduce the overall model size.

## Figures and Tables

**Table 1:** The specific settings for each layer of convolution blocks in EGAUNet. The layers in the Encoder include convolution and downsampling operations. The layers in the Decoder include upsampling and convolution operations.

Module	Layer Settings	Output Size
$Conv_{3\times 3}$	input 1; output 16	128 × 128
	1st layer: input 16; output 16	128 × 128
Encoder	2nd layer: input 16; output 24	64 × 64
	3rd layer: input 24; output 40	32 × 32
	4th layer: input 40; output 112	16 × 16
	5th layer: input 112; output 160	8 × 8
	1st layer: input 160; output 112	16 × 16
Decoder	2nd layer: input 224; output 40	32 × 32
	3rd layer: input 80; output 24	64 × 64
	4th layer: input 48; output 16	128 × 128
	5th layer: input 32; output 8	256 × 256
$Conv_{1\times 1}$	input 8; output class numbers	256 × 256

**Table 2:** Segmentation performance for different methods on the CHAOS T2SPIR dataset. The bold values indicate the best results.

Methods	Accuracy/%	Jaccard/%	Dice/%	Recall/%	Precision/%	Model Size/MB
U-Net [22]	99.241	81.4906	89.5248	85.5202	**94.5099**	93.268
U-Net++ [24]	99.273	81.4303	89.4413	86.9528	92.8613	99.540
DeepLabV3+ [37]	99.2	79.4703	87.543	86.5181	89.4392	85.653
DeepLabV3 [36]	99.1906	79.4616	87.8887	87.0126	90.4709	99.268
FPN [35]	99.0856	77.8403	86.6493	85.6116	88.491	88.374
PSPNet [34]	99.1414	77.0972	86.0903	84.7043	88.5407	81.896
PAN [39]	99.2099	79.111	87.3738	84.5139	91.2465	81.972
LinkNet [38]	90.2008	80.1587	88.5952	86.3175	91.9858	83.103
MA-Net [43]	99.2258	81.0168	89.2562	85.431	94.2853	121.307
TransUnet [32]	99.1924	80.4445	88.8427	86.5871	91.8494	401.783
Swin-Unet [33]	98.7621	80.8076	89.0952	87.2299	91.6879	159.942
**EGAUNet**	**99.2712**	**82.7481**	**90.3579**	**87.406**	94.0992	**13.687**

**Table 3:** Segmentation performance for different methods on the CHAOS T1DUAL dataset. The bold values indicate the best results.

Methods	Accuracy/%	Jaccard/%	Dice/%	Recall/%	Precision/%	Model Size/MB
U-Net [22]	98.7695	71.1743	81.8562	78.4352	87.4716	93.268
U-Net++ [24]	98.7683	72.5626	82.9347	80.0251	87.7143	99.540
DeepLabV3+ [37]	98.8295	70.8224	81.4241	81.0444	83.9067	85.653
DeepLabV3 [36]	98.8085	**72.6485**	83.0298	80.1389	90.5594	99.268
FPN [35]	98.7923	69.0447	79.4689	73.7402	88.5001	88.374
PSPNet [34]	98.6957	69.5157	80.6271	75.0415	**90.8589**	81.896
PAN [39]	98.8128	71.7114	82.6195	79.5785	88.3801	81.972
MA-Net [43]	98.7847	66.8831	77.8886	73.0551	85.8648	121.307
LinkNet [38]	98.6854	69.837	81.0474	78.4861	85.529	83.103
TransUnet [32]	98.8216	72.3102	83.06	**81.5979**	85.8489	401.783
Swin-Unet [33]	98.0135	68.7503	80.6263	77.4063	86.037	159.942
**EGAUNet**	**98.8731**	72.5935	**83.0767**	81.2664	86.1776	**13.687**

**Table 4:** Segmentation performance for different methods on the Brain MRI dataset. The bold values indicate the best results.

Methods	Accuracy/%	Jaccard/%	Dice/%	Recall/%	Precision/%
U-Net [22]	99.1921	70.0793	73.9475	73.2199	78.4849
U-Net++ [24]	99.222	70.684	73.9475	73.2199	78.4849
DeepLabV3+ [37]	99.2099	70.6161	73.8614	73.9679	77.5365
DeepLabV3 [36]	99.2552	71.5492	74.826	75.2761	77.5441
FPN [35]	99.264	71.0808	74.5197	74.3894	78.5029
PSPNet [34]	99.2063	70.1365	72.8361	73.1024	75.1314
PAN [39]	99.2041	70.0225	72.6904	72.513	76.0798
MA-Net [43]	99.2188	71.0317	74.0247	74.2834	77.2081
LinkNet [38]	99.2272	70.6377	74.4036	76.3936	77.3731
TransUnet [32]	99.2383	70.8217	73.8083	73.0028	77.9385
Swin-Unet [33]	**99.3482**	72.0496	75.797	75.9722	**79.0336**
**EGAUNet**	99.3067	**72.4873**	**75.8443**	**76.2431**	78.7209

**Table 5:** Segmentation performance for different methods on the Chest X-ray Masks and Labels dataset. The bold values indicate the best results.

Methods	Accuracy/%	Jaccard/%	Dice/%	Recall/%	Precision/%
U-Net++ [24]	98.1561	92.7387	96.1274	95.3239	97.2012
Linknet [38]	98.1659	92.7697	96.1624	95.322	97.235
PAN [39]	98.0818	92.44	95.9733	94.9952	**97.2226**
DeepLabV3+ [37]	**98.2201**	92.7515	96.1572	95.8293	96.6959
FPN [35]	98.1623	92.7661	96.1662	**95.9995**	96.542
TransUnet [32]	98.1856	92.786	96.1703	95.672	96.8906
Swin-Unet [33]	97.5572	92.6014	96.0786	95.3826	96.9877
**EGAUNet**	98.1811	**92.8105**	**96.1954**	95.6684	96.9125

**Table 6:** Performance of different attention mechanisms on the CHAOS T2SPIR dataset. The bold values indicate the best results.

Attention Mechanisms	Accuracy/%	Jaccard/%	Dice/%	Recall/%	Precision/%
CBAM [63]	99.2427	82.1369	89.8077	**87.7827**	92.881
GCnet [64]	98.2424	81.7871	89.6564	87.3808	92.5535
GAM [65]	98.9573	80.3427	88.6592	83.8494	**94.6779**
CCnet [66]	99.1597	81.7218	86.8582	82.4949	89.4902
SCSE [67]	99.2125	81.6256	89.6042	87.4182	92.6259
**GSCA**	**99.2712**	**82.7481**	**90.3579**	87.406	94.0992

**Table 7:** Ablation study on different modules in the proposed framework. The bold values indicate the best results.

GSCA	EMCB	GAL	Accuracy/%	Jaccard/%	Dice/%	Recall/%	Precision/%
×	×	×	99.081	80.9021	89.325	86.8637	91.021
✓	×	×	99.2439	81.3664	89.9033	87.0575	92.6582
×	✓	×	99.0958	81.1127	89.6771	87.067	91.3626
✓	✓	×	99.2615	81.4386	89.0968	87.2611	92.7406
✓	✓	✓	**99.2712**	**82.7481**	**90.3579**	**87.406**	**94.0992**

**Table 8:** Performance variation on different values of λ in the loss function. The bold values indicate the best results.

λ	Accuracy/%	Jaccard/%	Dice/%	Recall/%	Precision/%
0.03	99.2357	82.384	90.1588	87.2477	93.699
0.02	99.2359	82.5644	90.2338	89.0517	91.9896
0.015	99.2626	82.548	90.1384	**89.6218**	91.0939
0.01	**99.2712**	**82.7481**	**90.3579**	87.406	**94.0992**
0.005	99.1796	81.1614	89.3579	88.3616	91.0004

**Table 9:** Segmentation performance with/without (i.e., w/o) residual connections (i.e., Res). The bold values indicate the best results.

Methods	Accuracy/%	Jaccard/%	Dice/%	Recall/%	Precision/%
w/o Res	99.197	81.0099	89.1963	87.0337	91.9229
Res	**99.2712**	**82.7481**	**90.3579**	**87.406**	**94.0992**

**Table 10:** Computational costs for different models. The bold values indicate the best results.

Methods	Model Size/MB	GFLOPs	Iter/s
U-Net [22]	93.268	124.13	1.16
U-Net++ [24]	99.540	293.36	1.15
DeepLabV3+ [37]	85.653	124.77	1.13
DeepLabV3 [36]	99.268	435.41	1.30
FPN [35]	88.374	108.30	1.13
PSPNet [34]	81.896	36.20	**1.10**
PAN [39]	81.972	117.60	1.13
MA-Net [43]	83.103	85.58	1.13
LinkNet [38]	121.307	132.19	1.13
TransUnet [32]	401.783	615.67	1.42
Swin-Unet [33]	159.942	143.09	1.19
**EGAUNet**	**13.687**	**15.26**	1.22

## Data Availability

The datasets used in this study can be downloaded from the urls in their official websites. The code is accessible from the corresponding author upon reasonable request.
